# Phase‐Amplitude Coupling in Sleep EEG—Stable Trait or Shaped by Experience? (Commentary on Cross et al., 2025)

**DOI:** 10.1111/ejn.70204

**Published:** 2025-07-18

**Authors:** Niels Niethard

**Affiliations:** ^1^ Institute of Medical Psychology and Behavioral Neurobiology University of Tübingen Tübingen Germany; ^2^ Department of Cognitive Sciences University of California Irvine California USA; ^3^ Institute for Diabetes Research and Metabolic Diseases of the Helmholtz Center Munich at the University Tübingen (IDM) Tübingen Germany

The consolidation of newly encoded memories into long‐term storage critically depends on plasticity processes during sleep. It is assumed that memory representations are facilitated by repeated reactivation of neuronal firing patterns during sleep, which promotes synaptic plasticity and thereby strengthens memory traces. A growing body of evidence shows that such memory reactivations occur during specific oscillatory patterns in the EEG that are unique to sleep (Brodt et al. 2023).

In particular, cortical slow oscillations (SOs) and thalamocortical spindles have been consistently associated with enhanced memory consolidation during sleep. SOs are large‐amplitude, low‐frequency fluctuations lasting between 0.5 and 2 s, reflecting transitions between cortical down states (neuronal silence due to hyperpolarization) and up states (neuronal depolarization and increased excitability). Sleep spindles, another hallmark of NREM sleep, are brief bursts of activity in the 11 to 16 Hz frequency range characterized by waxing and waning amplitudes. Crucially, the precise temporal coupling between SOs and spindles has emerged as a key mechanism supporting synaptic plasticity and the stabilization of memory traces during NREM sleep (Schreiner et al. 2021; Brodt et al. 2023; Staresina 2024). Prior research has shown that aging alters this SO‐spindle coupling, and that such alterations are associated with cognitive decline and impaired memory performance (Helfrich et al. 2017; Muehlroth et al. 2019; Hahn et al. 2020). However, it remains unclear whether SO‐spindle coupling represents a stable, trait‐like feature of an individual's sleep architecture or whether it is an adaptive mechanism that can vary depending on recent experiences, such as memory encoding.

To investigate whether SO‐spindle coupling is influenced by prior learning, Cross et al. (2025) conducted a study involving 41 participants who underwent overnight polysomnographic recordings. Participants experienced two experimental conditions: one night following a word‐pair learning task and a control night without any preceding learning. The study also manipulated learning load across groups—participants either learned 40 or 120 word pairs—and introduced a performance‐based criterion for one of the 40‐word‐pair groups. Specifically, the criterion group was required to achieve at least 60% correct recall to proceed, whereas the other 40‐word and 120‐word groups were exposed to the word pairs twice, regardless of recall performance. While Cross and colleagues observed a correlation between memory performance and the phase of SO‐spindle coupling in the group that met the learning criterion, they did not find any significant differences in SO‐spindle coupling between the learning and control nights across any of the experimental conditions. Notably, they also reported a strong correlation between spindle‐band power and the preferred phase of SO‐spindle coupling in frontal EEG channels (Cross et al. 2025)—a pattern that has previously been shown to differ between younger and older adults (Helfrich et al. 2017). Yet considerable variability remains even within the same age group, suggesting that additional, potentially modifiable factors may also play a significant role.

Thus, a central question remains: is SO‐spindle coupling a stable, trait‐like feature that reflects an individual's inherent capacity for memory consolidation, or is it a flexible, state‐dependent process that can change dynamically? While Cross and colleagues did not find evidence that pre‐sleep learning affects SO‐spindle coupling, this does not definitively rule out such a relationship. Notably, even during the control night, participants would have encoded a large amount of information throughout the day that still required consolidation during sleep. The total amount of information encoded in daily life likely far exceeds that encoded in a word‐pair learning task.

What other factors might influence SO‐spindle coupling? Recent findings suggest that coupling strength and precision—quantified as the proportion of coupled spindles and their temporal alignment with SOs—are negatively associated with next‐day fasting glucose levels (Vallat et al. 2023). Importantly, these correlations remained significant after controlling for variables such as age, sex, race, BMI, hypertension, sleep apnea severity, and sleep duration, but disappeared when diabetes status was included as a covariate. This suggests that metabolic status and eating patterns—factors with typically high intra‐individual stability— shape the temporal structure of sleep oscillations.

Indeed, experimental data from adult rats support the idea that eating behavior affects sleep oscillations. In a recent study, systemic glucose vs. vehicle administration and short‐term fasting vs. ad libitum food access (6 h) were used to manipulate metabolic states prior to sleep (Lun et al. 2025). Fasting led to a significant increase in the density of SOs and sleep spindles, as well as a higher rate of their co‐occurrence. It also shifted the timing of their phase‐amplitude coupling: after fasting, spindles occurred later, aligning more closely with the SO upstate—a configuration previously associated with enhanced memory consolidation during sleep (Schreiner et al. 2021). Additional LFP recordings from the CA1 area of the hippocampus showed that fasting increased ripple density compared to ad libitum access to food. In contrast, intraperitoneal glucose injection increased spindle density but did not affect SOs, SO‐spindle coupling, or hippocampal ripples. Notably, these changes occurred without affecting overall sleep architecture, as NREM and REM sleep durations remained stable across conditions.

Together, these findings support the view that while factors like age set a general framework for SO‐spindle coupling, experience‐dependent influences such as fasting can modulate its temporal dynamics—indicating that SO‐spindle coupling is not entirely a stable trait, but is also shaped by experience, with potential consequences for how effectively memories are consolidated during sleep.

Future studies are needed to dissect the circuit and network mechanisms that govern the precise timing of SOs and spindles. Emerging evidence from rodent studies indicates that, on slow timescales spanning tens of seconds to minutes, NREM sleep comprises recurring substates characterized by fluctuating levels of neuromodulators such as serotonin, acetylcholine, and norepinephrine—all of which are implicated in memory processing (Sulaman et al. 2024). How these slow neuromodulatory dynamics influence SO–spindle coupling remains largely unknown and warrants further investigation.

## Conflicts of Interest

The authors declare no conflicts of interest.

## Peer Review

The peer review history for this article is available at https://www.webofscience.com/api/gateway/wos/peer‐review/10.1111/ejn.70204.
